# A principal component analysis of the post-secondary student stressors index in a sample of Ontario students

**DOI:** 10.1371/journal.pmen.0000431

**Published:** 2025-09-10

**Authors:** Sarah Kuburi, BingSen Wang, Chloe A. Hamza, Brooke Linden

**Affiliations:** 1 Department of Applied Psychology and Human Development, Ontario Institute for Studies in Education, University of Toronto, Ontario, Canada; 2 Health Services and Policy Research Institute, Queen’s University, Kingston, Ontario, Canada; PLOS: Public Library of Science, UNITED KINGDOM OF GREAT BRITAIN AND NORTHERN IRELAND

## Abstract

Post-secondary students report high levels of stress and mental health challenges. Identifying the key sources of stress can guide efforts to mitigate the most significant stressors and better support students’ mental health. The Post-Secondary Student Stressors Index (PSSI), a 46-item inventory, was designed to comprehensively assess stressors specific to post-secondary students. However, its dimensional structure and its association with mental health indices require further validation. In the present study, a Principal Component Analysis was conducted to examine the structure of the PSSI, and the associations between the resulting stressor components and several mental health indices. Participants included 1,214 first-year students (*Mage* = 18.14 years, SD = 0.95), who completed the PSSI, as well as measures of perceived stress, depressive symptoms, and anxiety symptoms. Results suggested 12 components that accounted for 42 of the original 46 items, with most components demonstrating moderate to high internal consistency (α = .70 -.90). Correlational analyses revealed positive associations between the PSSI components and measures of perceived stress, depressive symptoms, and anxiety symptoms, although components with lower endorsements were associated with weaker correlations. By identifying valid and reliable components of the PSSI, this study facilitates the use of specific stressor components in future research on student stress and mental health.

## Introduction

Emerging adulthood, defined as the period between ages 18 and 25 years, often coincides with post-secondary education enrollment in Canada [1]. In addition to the inherent developmental challenges of this period, such as identity formation and the pursuit of independence [2], the transition to university introduces unique stressors. Students must navigate new academic demands, and adjust to unfamiliar social environments [3]. For many, living away from home and establishing new support networks while attending post-secondary education intensifies feelings of stress [4]. Additionally, students continue to navigate the lingering academic and social disruptions from the COVID-19 pandemic [5,6]. Recent data from the National College Health Survey reports that approximately 78% of students report moderate to high stress levels during their university years, with 35% diagnosed with anxiety and 25% with depression [7].

Prior research on student stress has often overlooked the need to examine specific stressors and their differential impacts on mental health within the post-secondary environment [8]. Stressor assessments used in this context are typically broad and generalized, limiting their ability to address the unique circumstances under which student samples experience stressors. Most assessments that aim to examine stressors specific to students focus primarily on academic stress, despite evidence suggesting that students face a wide range of stressors beyond the academic domain [9,10]. Additionally, assessments that intend to capture a range of post-secondary stressors often neglect campus-specific issues, such as challenges related to post-secondary adjustment, and sociocultural diversity (e.g., discrimination, sexual harassment), largely due to the limited scope of research on diverse student groups [11]. Furthermore, many existing stress measures are outdated, containing items that no longer reflect the current realities of student experiences [12].

Existing stress measures often do not fully capture the range of stressors students experience due to the limited involvement of students from diverse backgrounds in the development of these tools [8]. Stress assessments that are not designed in collaboration with students tend to focus on a narrow set of stressors, overlooking the variety of experiences across diverse student samples [13]. Additionally, current measures often do not account for the varying severity of stressors, though this is crucial for understanding how different stressors affect students’ mental health, as not all stressors have the same impact [14]. Consequently, existing post-secondary stress measures are limited in capturing the wide range of stressors experienced by students, as well as the most impactful stressors affecting student mental health. More accurate identification of these stressors and their varying levels of severity can help tailor campus mental health initiatives to better meet the unique needs of their student samples.

The Post-Secondary Student Stressors Index (PSSI) is a recently developed instrument designed to comprehensively assess the range of stressors experienced by post-secondary students [8]. The PSSI consists of 46 items that capture a wide range of stressors and their severity, including those related to academics, the learning environment, campus culture, interpersonal relationships, and personal challenges. The PSSI was developed using a ‘for-students-by-students’ approach, ensuring that the measure accurately reflects the experiences of post-secondary students by actively involving students as subject matter experts in its design. Empirical validation has demonstrated robust psychometric properties, confirming its content validity and test-retest reliability [8]. Further, validation studies affirm the PSSI’s internal structure and reveal significant correlations with related constructs, reinforcing its capacity to capture the wide scope of stressors affecting post-secondary students [8,10].

Although the PSSI addresses several gaps in existing stress measures, limitations remain in its ability to assess the impact of specific stressors on student mental health. The grouping of items into broad categories is based primarily on content area rather than psychometric evidence, which restricts the development of statistically valid and reliable components. This limitation reduces the capacity to examine how different types of stressors impact student mental health and increases the risk of type I errors when associations between multiple individual items and mental health outcomes are analyzed independently [15]. Dimensionality testing could address this limitation by identifying distinct components among the 46 items, enabling a more precise categorization of stressors to examine their differential effects on mental health indices [16]. Although efforts have been made to create a short form of the measure (Brief-PSSI; [17]), further refinement of the full version may help reduce item redundancy while maintaining the breadth of stressor coverage necessary for a comprehensive assessment of student experiences.

Principal Component Analysis (PCA) is a statistical technique commonly used to reduce the complexity of large datasets while retaining as much of the original information as possible [18]. In the context of the PSSI, a PCA can be applied to reduce the dimensionality of the 46 stressor items by identifying underlying components that capture the most relevant stressors. This results in fewer stressor categories, making the data more manageable and interpretable for further analysis. Once these components are established, internal consistency analysis can also be conducted to assess the reliability of these components in accurately measuring specific stressor categories [19]. Furthermore, grouping related stressors into distinct components allows for the exploration of how these categories correlate with mental health indices, providing additional evidence of their validity and reliability. This process then enables researchers to investigate the associations between reliable stressor categories and various mental health indices, offering valuable insights into the unique ways stressors impact students’ mental health and well-being [20].

To date, only one study has conducted a PCA on the PSSI, which was carried out at a university in Manitoba, Canada [21]. The results indicated that the 46 PSSI items were reduced to 10 components, encompassing 40 items, which demonstrated moderate to strong internal reliability. However, further research is needed across different regions of Canada to confirm the dimensionality of the PSSI among diverse student samples, as stressor components may vary based on regional and experiential differences (e.g., students in urban Manitoba may experience different stressors than those in urban Ontario). Additionally, the study did not examine the associations between the PSSI components and mental health indices, such as perceived stress, depressive symptoms, and anxiety symptoms. Exploring these associations is crucial, as stressors are inherently linked to mental health indices [22] and strong correlations between the PSSI components and these indicators would further validate the scale’s reliability in capturing the psychological impact of stressors among post-secondary students.

### Current study

Previous research on the PSSI has been limited in identifying the underlying components of the measure and their reliability across various post-secondary student samples. Although a PCA has been applied to the PSSI in a sample of students in Manitoba [21], a significant gap also remains in understanding how these components correlate with mental health indices such as perceived stress, depressive symptoms, and anxiety symptoms. The aim of the present study is to address these gaps by identifying the components of the PSSI using a PCA with a sample of students from a large urban university in Ontario, Canada. The reliability of the identified components was also assessed, and correlations with mental health indices were examined. By exploring these associations, the study will provide a more comprehensive understanding of how the PSSI relates to student mental health indices. Identifying reliable components will not only validate the PSSI but also enhance its utility, enabling the use of specific components as predictors in future research to better understand the impact of distinct stressors on post-secondary student mental health and well-being.

## Materials and methods

### Ethics statement

This study received ethics approval from the university’s Research Ethics Board (Protocol Number 34625), and prior to participation, all individuals provided written informed consent.

### Participants

The present sample consisted of 1,214 university students (*M*age = 18.14 years, SD = 0.95) who participated in an ongoing longitudinal study on stress and coping during the transition to university, conducted between October 7, 2022, and June 1, 2023. The participants included in the current analysis completed the baseline survey of this larger project, which was administered between October 7 and December 2, 2022. Eligibility criteria required participants to be between 17 and 25 years of age, enrolled in their first year, and residing in or near the university’s city to ensure access to campus resources if needed.

### Procedures

Participants were recruited through multiple channels, including posters, program and class email distribution lists, student organizations and clubs, in-class announcements, and social media advertisements. Recruitment efforts were carried out across all three campuses of the university, recruiting first-year students across all programs. Posters were placed in designated areas where advertisements were permitted on each campus. In-class announcements were made in courses with at least 50 first-year university students enrolled, resulting in 32 classroom presentations. In cases where instructors did not permit in-class announcements, many agreed to share the study on the course website or via email with their students; these alternative forms of dissemination occurred in an additional 31 classes. All available first-year student email distribution lists and student organizations/clubs were contacted. In total, 16 recruitment emails were sent via listservs, one student organization agreed to distribute the study, and recruitment posts were shared in 9 relevant Facebook groups. Each recruitment effort was attempted three times, and the numbers reported above reflect the outcomes after these three attempts.

Interested individuals were instructed to contact the research team via email. Eligibility was assessed by the research coordinator upon initial contact (via email) and was confirmed through institutional email verification to ensure participants were registered students at the university. Students who met the inclusion criteria were assigned a unique participant identifier to complete the baseline survey hosted on Qualtrics. To minimize participant discomfort, participants were given the option to skip any survey questions, access a list of local mental health resources through a “Feeling Distressed” button any time during or after the survey, and withdraw from the study at any time. Participants also reflected on one good thing that happened to them that day at the end of each survey, as this has been shown to be an effective mood induction [23]. To encourage participation, compensation was provided in the form of electronic gift cards, allowing participants to select their preferred vendor from a list. Participants received $15 for completing the baseline survey.

## Measures

### Demographics

To assess the demographic characteristics of the sample, participants were asked to provide information on the following: age, gender (man, woman, nonbinary, transgender woman, transgender man, unsure, or a free response option), ethnicity (selected from a list where students could choose multiple options), student residency (domestic or international), student status (full-time or part-time), faculty (i.e., department or academic division), marital status (single, dating, in a serious relationship, married, or a free response option), sexual orientation (straight, lesbian, gay, bisexual, asexual, queer, unsure, or a free response option), campus residency (living on or off campus), living arrangements (living alone, with roommates/friends, with parents, with other family members, with a partner, or a free response option), hours of paid work (0 hours, 1–5 hours, 6–10 hours, 11–20 hours, 20 + hours), and parental education level, summarized into the highest education attained among parents (e.g., grade school, high school diploma, college or trade school, some university, undergraduate degree, or postgraduate degree).

### Post-secondary student stressors

Participants completed the PSSI, a comprehensive tool designed to assess stressors specifically experienced by students in post-secondary education [24]. The PSSI was developed using a ‘for-students-by-students’ approach and includes 46 items designed to broadly assess academic, learning environment, campus culture, interpersonal, and personal stressors. Participants rated the severity of each stressor on a four-point Likert scale from 1 (*Not stressful*) to 4 (*Extremely stressful*). Participants were also given the option to select certain stressors as ‘not applicable’ (i.e., stressors that had never occurred to them), and these responses were subsequently coded as zeros. The psychometric properties of the PSSI have been well established among post-secondary student samples in Canada [10]. In the present study, internal consistency was assessed using Cronbach’s alpha, with results for each measure reported in the preliminary results section.

### Perceived stress

Participants’ perceived stress was measured using the Perceived Stress Scale - 10 (PSS-10) [25], which assesses the degree to which situations in a participant’s life are subjectively perceived as stressful. The PSS-10 consists of 10 items that inquire about participants’ feelings and thoughts over the past month. Example items include “How often have you been upset because of something that happened unexpectedly?” and “How often have you felt that things were going your way?” Participants responded using a five-point Likert scale ranging from 0 (*Never*) to 4 (*Very often*). One item from the PSS-10 (“How often have you felt difficulties were piling up so high that you could not overcome them?”) was missing from this study due to human oversight. The omission of this item may slightly impact the measure’s comprehensiveness, though internal consistency remained robust, suggesting minimal impact on overall measurement validity. To calculate the final score, the four positively worded items were reverse-scored, and the mean of the remaining nine items was computed, with higher scores indicating greater perceived stress.

### Depressive symptoms

Participants’ depressive symptoms were assessed using the Centre for Epidemiological Studies Depression Scale-Revised (CESD-R) [26]. The CESD-R consists of 20 items (e.g., “I could not focus on the important things”*;* “Nothing made me happy”) that measure depressive symptoms based on the criteria outlined in the Diagnostic and Statistical Manual of Mental Disorders, Fifth Edition [27]*.* Due to ethical constraints, two items (“I wished I were dead” and “I wanted to hurt myself”) were excluded from this study. These items were removed in accordance with institutional ethics guidelines, which recommended avoiding direct references to self-harm in unsupervised online surveys to minimize participants distress in a non-clinical context without immediate support. However, truncated versions of the CESD-R are documented in the literature and are considered valid and reliable measures of depressive symptoms, although they also exclude items related to self-harm (e.g., the CESD-R-10) [28]. Participants rated each item based on symptom frequency, using a five-point Likert scale ranging from 0 (*Not at all or less than one day last week*) to 4 (*Nearly every day for two weeks*). A total score was calculated as the mean of the remaining eighteen items, with higher scores indicating greater depressive symptom severity.

### Anxiety symptoms

The Generalized Anxiety Disorder - 7 (GAD-7) scale [29] was used to assess the likelihood of generalized anxiety disorder occurring in participants. Participants rated how often they experienced each symptom (e.g., “Feeling nervous, anxiety, or on edge”; “Not being able to stop or control worrying”) over the past two weeks on a four-point Likert scale ranging from 0 (*Not at all*) to 3 (*Nearly every day*). If any symptoms were endorsed, participants also indicated how much these symptoms interfered with their daily functioning in occupational, home, and social domains, using a four-point Likert scale ranging from 0 (*Not difficult at all*) to 3 (*Extremely difficult*). A total score was calculated as the mean of the seven items, with higher scores indicating a greater likelihood of generalized anxiety disorder.

### Data analytic strategy

Descriptive analyses were first performed to summarize the demographic characteristics of the sample, as well as the other measures. The inter-rater reliability of the measures was also examined. Next, a PCA was conducted to reduce the dimensionality of the PSSI by transforming the original variables into a smaller set of components, retaining the maximum amount of information as possible. The Kaiser-Meyer-Olkin (KMO) test and Bartlett’s Test of Sphericity were utilized to assess the suitability of the present data for a PCA by evaluating the correlations among the variables. Components were extracted based on eigenvalues greater than 1, with factor loadings suppressed at 0.4 for each component, using direct oblimin oblique rotation. The rotated factor loadings and corresponding items for each component were subsequently reviewed for interpretation, and these components were evaluated for internal consistency using Cronbach’s alpha. A follow-up sensitivity analysis was also conducted to examine if the PCA results varied when the factor loading suppression threshold was lowered to 0.3 [30]. Components with more factor loadings due to the lower suppression threshold were subsequently reassessed for differences in internal consistency using Cronbach’s alpha.

Missing data among the PSSI items were extremely minimal, ranging from 0% to 0.006% per item (i.e., a minimum of 0 and a maximum of 7 missing responses dependent on the item, with most missing responses in the lower range). Missing data were addressed using listwise deletion, which is considered appropriate when the proportion of missing values is extremely low [31,32]. Finally, correlations among the PSS-10, CESD-R, GAD-7, and the PSSI components were explored to examine the associations between mental health symptoms and components of stressors. The PSSI total score was excluded from the correlational analyses, as it is not considered appropriate for indices due to the large number of items, which would inflate Cronbach’s alpha [33]. All analyses were performed using SPSS Statistics version 29.0.

## Results

### Sample characteristics

The majority identified as women (71.1%), while 25% identified as men and most participants self-identified as White, South Asian, or East Asian (79.3%). Additional demographic characteristics are presented in Table 1.

**Table 1:** Sample characteristics.

Demographics	*N* (%)
Gender	
Woman	863 (71.1%)
Man	304 (25%)
Transgender Woman, Transgender Man, Unsure, or a Free Response Option	47 (3.9%)
Ethnicity	
White	255 (21%)
East Asian	437 (36%)
South Asian	271 (22.3%)
Black	82 (6.8%)
Arab/West Asian	83 (6.8%)
Latin American/Hispanic	61 (5%)
Indigenous, Southeast Asian, Filipino, West Indian	156 (12.8%)
Student Residency	
Domestic	852 (70.2%)
International	360 (29.7%)
Missing	2 (.2%)
Student Status	
Full Time	1196 (98.6%)
Part Time	15 (1.2%)
Missing	3 (.2%)
Faculty	
Humanities & Social Sciences	442 (36.4%)
Life Sciences	372 (30.6%)
Commerce & Management	131 (10.8%)
Physical & Mathematical Sciences	92 (7.6%)
Computer Sciences, Engineering, Kinesiology & Physical Education, Music & Architecture	85 (7%)
Free Response Option	91 (7.5%)
Missing	1 (.1%)
Marital Status	
Single	937 (77.2%)
Dating	182 (15%)
In a Serious Relationship or Married	93 (7.6%)
Missing	2 (.2%)
Sexual Orientation	
Straight/Heterosexual	894 (73.6%)
Bisexual	154 (12.7%)
Lesbian, Gay, Asexual, Queer, Unsure, or a Free Response Option	165 (13.6%)
Missing	1 (.1%)
Campus Residency	
On Campus	539 (44.4%)
Off Campus	673 (55.4%)
Missing	2 (.2%)
Living Arrangements	
Living Alone	198 (16.3%)
Living with Roommates/Friends	507 (41.8%)
Living with Parents	417 (34.3%)
Living with Other Family, A Partner, or a Free Response Option	90 (7.4%)
Missing	2 (.2%)
Hours of Paid Work	
0 Hours	901 (74.2%)
1-5 Hours	104 (8.6%)
6-10 Hours	80 (6.6%)
11-20 + Hours	95 (7.8%)
Missing	34 (2.8%)
Parental Education	
Post Graduate Degree	491 (40.4%)
University Undergraduate Degree	376 (31%)
College or Trade School	107 (8.8%)
High School Diploma or GED	71 (5.8%)
Grade School or Some University	61 (5.1%)
Don’t Know or Unsure	107 (8.8%)
Missing	1 (.1%)

**Notes.** N = sample size.

### Preliminary results

**Fig 1:**
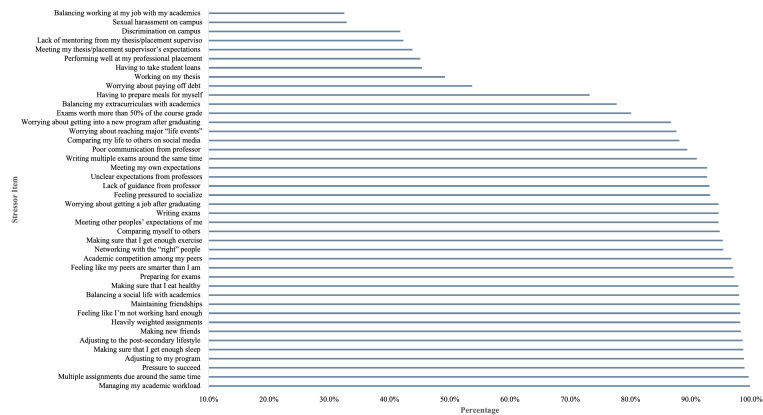
Percentage of participants endorsing each stressor item.

The most commonly endorsed stressor among students in this sample was managing their academic workload, reported by 99.8% of participants. The percentages of participants endorsing the remaining stressor items are presented in Fig 1. Participants reported a mean score of 2.11 (SD = 0.59) on the PSS-10, 1.23 (SD = 0.83) on the CESD-R, and 1.35 (SD = 0.83) on the GAD-7. The mental health measures used in this study demonstrated good to excellent internal consistency, despite some missing items in both the CESD-R and PSS-10, as assessed by Cronbach’s alpha. Specifically, the CESD-R (α = .93) and GAD-7 (α = .91) showed excellent internal consistency, while the PSS-10 demonstrated good internal consistency (α = .79).

### Primary results

The adequacy of the sample was confirmed, with a KMO value of .908 indicating excellent sampling adequacy, and a significant Bartlett’s Test of Sphericity (χ²(1035) = 23,353.65, *p* < .001), supporting the suitability of the data for factor analysis. In the PCA analysis, 12 components were identified, comprising 42 of the 46 items from the PSSI (see Table 2). Component 1 (Academic Comparisons) comprised eight items reflecting social comparison, academic pressure, and the expectations students place on themselves and perceive from others. Component 2 (Thesis Achievements) included four items focused on expectations, mentoring, and performance in relation to thesis work and professional placements. Component 3 (Interpersonal Relationships) consisted of five items describing challenges in forming and maintaining social connections, navigating networking expectations, and balancing social and academic commitments. Component 4 (Finances) included two items addressing concerns related to student debt and reliance on loans. Component 5 (Professor/Advisor Interactions) encompassed three items detailing difficulties in professor-student interactions, including unclear expectations, poor communication, and lack of guidance. Component 6 (Exams) comprised four items related to the stress associated with exam preparation and examination periods. Component 7 (Healthy Lifestyle) consisted of four items regarding the maintenance of a healthy lifestyle, emphasizing physical activity, nutrition, sleep, and meal preparation. Component 8 (Discrimination/Harassment) included two items concerning experiences of discrimination and sexual harassment on campus. Component 9 (Post-Secondary Adjustment) consisted of two items reflecting the challenges of adapting to the academic program and the post-secondary lifestyle. Component 10 (Academic Workload) included three items relating to the management of academic responsibilities, including overlapping assignment deadlines, heavily weighted assignments, and overall workload management. Component 11 (Balancing Responsibilities) encompassed two items focused on balancing work and extracurricular activities alongside academic demands. Finally, Component 12 (Future Worrying) consisted of three items addressing concerns regarding post-graduation plans, including further education, life milestones, and job prospects. Four items did not load onto any component as their factor loadings were below the 0.4 threshold. These items were: meeting with a professor, feeling guilty about taking time for hobbies and interests, managing a high GPA, and receiving a bad grade.

**Table 2:** Principal components, items by each component, and component loadings.

Components	Component Loadings
** Items**
Academic Comparisons	
** **1) Comparing myself to others	.729
** **2) Feeling like my peers are smarter than I am	.704
** **3) Feeling like I’m not working hard enough	.624
** **4) Comparing my life to others on social media	.589
** **5) Meeting other peoples’ expectations of me	.548
** **6) Academic competition among my peers	.542
** **7) Pressure to succeed	.515
** **8) Meeting my own expectations	.487
Thesis Achievements	
** **1) Meeting my thesis/placement supervisor’s expectations	.925
** **2) Lack of mentoring from my thesis/placement supervisor	.881
** **3) Working on my thesis	.873
** **4) Performing well at my professional placement (i.e., practicum, clerkship, etc.)	.777
Interpersonal Relationships	
** **1) Making new friends	.841
** **2) Maintaining friendships	.807
** **3) Networking with the “right” people	.753
** **4) Feeling pressured to socialize	.746
** **5) Balancing a social life with academics	.415
Finances	
** **1) Worrying about paying off debt	.909
** **2) Having to take student loans	.908
Professor/Advisor Interactions	
** **1) Unclear expectations from professor	.910
** **2) Poor communication from professor	.875
** **3) Lack of guidance from professor	.850
Exams	
** **1) Writing multiple exams around the same time	.895
** **2) Writing exams	.814
** **3) Exams worth more than 50% of course grade	.757
** **4) Preparing for exams	.659
Healthy Lifestyle	
** **1) Making sure that I get enough exercise	.847
** **2) Making sure that I eat healthy	.806
** **3) Making sure that I get enough sleep	.718
** **4) Having to prepare meals for myself	.406
Discrimination/Harassment	
** **1) Discrimination on campus	.899
** **2) Sexual harassment on campus	.891
Post-Secondary Adjustment	
** **1) Adjusting to my program	.691
** **2) Adjusting to the post-secondary lifestyle	.676
Academic Workload	
** **1) Having multiple assignments due around the same time	-.811
** **2) Heavily weighted assignments	-.668
** **3) Managing my academic workload	-.555
Balancing Responsibilities	
** **1) Balancing working at my job with my academics	-.769
** **2) Balancing my extracurriculars with academics	-.741
Future Worrying	
** **1) Worrying about getting into a new program after graduating	.654
** **2) Worrying about reaching major “life events” (i.e., buying a house, marriage, children)	.606
** **3) Worrying about getting a job after graduating	.569

Notes. Items that did not load onto components above the 0.4 threshold include meeting with professor, feeling guilty about taking time for my hobbies/interests, managing a high GPA, and receiving a bad grade.

The internal consistency was examined for each component using Cronbach’s alpha coefficients to assess the degree to which items within a component measure the same underlying construct. The alpha values for each component are presented in Table 3. Cronbach’s alpha coefficients ranged from .70 to .90, demonstrating moderate to high internal consistency across the components. However, Component 11 (Balancing Responsibilities) had a lower Cronbach’s alpha coefficient of .52, indicating inadequate internal consistency. As a result, this component was excluded from the following correlational analyses.

**Table 3:** Reliability coefficients per component.

Component	Cronbach’s Alpha
Academic Comparisons	.87
Thesis Achievements	.90
Interpersonal Relationships	.83
Finances	.86
Professor/Advisor Interactions	.85
Exams	.80
Healthy Lifestyle	.70
Discrimination/Harassment	.77
Post-Secondary Adjustment	.74
Academic Workload	.71
Balancing Responsibilities	.52
Future Worrying	.73

### Sensitivity analysis

The present study applied a factor loading threshold of 0.4, however, prior research suggests that a threshold of 0.3 may also be acceptable [30]. To assess the impact of this criterion, a sensitivity analysis was conducted to determine whether items that did not load onto any components at the 0.4 threshold would do so at the 0.3 threshold. Results indicated that three of the four previously unassigned items loaded onto components at the 0.3 level. Specifically, feeling guilty about taking time for my hobbies/interests (-.339) loaded onto the Balancing Responsibilities component. To examine whether this addition improved the component’s reliability, Cronbach’s alpha was recalculated, yielding a slight increase in internal consistency from α = .52 to α = .55, though it remained below an acceptable threshold. Additionally, managing a high GPA (.381) and receiving a bad grade (.362) loaded onto the Future Worrying component. When recalculating internal consistency, there was a slight increase from α = .73 to α = .74. Given these findings, the original PCA with a 0.4 threshold was deemed the most appropriate, as lowering the criterion did not significantly improve the structure or reliability of the components.

### Correlational analysis

The correlations among the PSS-10, CESD-R, GAD-7, and the components of the PSSI were examined to assess the associations between mental health indices, perceived stress, and the components of the PSSI. The correlation coefficients, presented in Table 4, ranged from .037 to .784. Notably, the components of the PSSI were generally moderately and significantly positively correlated with the PSS-10, CESD-R, and GAD-7, indicating that higher scores in these specific components were linked to greater perceived stress, depressive symptoms, and anxiety symptoms. It is worth noting that the Thesis Achievements and Discrimination/Harassment components exhibited lower correlation coefficients, ranging from .037 to .148. This may be attributed to the relatively low endorsement of items within these components (i.e., fewer than 50% of students reported endorsing the stressor items within these components, as shown in Fig 1). This likely contributed to the lower correlations among these components, mental health, and perceived stress indices.

**Table 4:** Correlations among the PSS-10, CESD-R, GAD-7, and PSSI components.

Measures	1	2	3	4	5	6	7	8	9	10	11	12	13	14
1) PSS-10	–													
2) CESD-R	.661**	–												
3) GAD-7	.686**	.752**	–											
4) Academic Comparisons	.526**	.512**	.536**	–										
5) Thesis Achievements	.062*	0.037	.104**	.132**	–									
6) Interpersonal Relationships	.395**	.377**	.373**	.508**	.097**	–								
7) Finances	.226**	.266**	.272**	.285**	.174**	.168**	–							
8) Professor/Advisor Interactions	.264**	.223**	.277**	.337**	.268**	.218**	.170**	–						
9) Exams	.288**	.222**	.302**	.357**	.308**	.183**	.192**	.309**	–					
10) Healthy Lifestyle	.313**	.371**	.362**	.416**	.155**	.267**	.258**	.226**	.226**	–				
11) Discrimination/Harassment	.101**	.136**	.148**	.169**	.247**	.180**	.133**	.220**	.176**	.133**	–			
12) Post-Secondary Adjustment	.414**	.357**	.392**	.518**	.185**	.361**	.222**	.240**	.320**	.309**	.103**	–		
13) Academic Workload	.411**	.364**	.413**	.516**	.220**	.366**	.199**	.331**	.448**	.312**	.112**	.451**	–	
14) Future Worry	.372**	.365**	.404**	.495**	.218**	.318**	.418**	.306**	.333**	.319**	.152**	.319**	.344**	–

***Notes.*** PSS-10 = Perceived Stress Scale, CESD-R = Centre for Epidemiological Studies Depression Scale-Revised, GAD-7 = Generalized Anxiety Disorder scale, PSSI = Post-Secondary Student Stressors Index.

* *p* < .05; ** *p* < .01.

## Discussion

The aim of the present study was to identify the underlying components of the PSSI using a PCA in a sample of university students at a large urban university in Ontario. The study also assessed the reliability of the derived components and examined their associations with mental health indices, including perceived stress, depressive symptoms, and anxiety symptoms. Twelve components were identified using a PCA, representing 42 out of the 46 original PSSI items. Reliability analyses revealed moderate to high internal consistency across most components, apart from Balancing Responsibilities, which exhibited inadequate reliability. Sensitivity analyses indicated that a 0.4 threshold was the most appropriate criterion, as lowering this threshold did not meaningfully improve the component structure or reliability. Lastly, most PSSI components were moderately to strongly correlated with perceived stress, depressive symptoms, and anxiety symptoms. However, less frequently endorsed components (e.g., Thesis Achievements and Discrimination/Harassment) showed weaker associations with mental health indices.

A previous study conducted at the University of Manitoba, which also employed a PCA to derive components of the PSSI among university students [21], identified 10 components, which differs from the 12 components found in the present study (see S1 Appendix). Despite the difference in the number of components, 10 components were consistent across both studies, encompassing the same stressor categories: Academic Comparison, Thesis Achievements, Exams, Healthy Lifestyle, Finances, Professor/Advisor Interactions, Interpersonal Relationships, Harassment/Discrimination, Post-Secondary Adjustment, and Academic Workload. Additionally, item loadings were largely consistent across these components, with minimal variability in the specific items that loaded onto each component between the two studies. These findings suggest that the dimensionality of the PSSI may be mostly stable across different student samples, supporting its potential generalizability and utility in comprehensively assessing student stress across diverse post-secondary settings [17].

Two additional components, Balancing Responsibilities and Future Worrying, emerged in the present sample, although they were not identified in the PCA conducted at the University of Manitoba. The naming of these components was based on the interpretation of the primary themes reflected in the items that loaded onto each component. Although the Future Worrying component demonstrated good internal consistency, it is important to highlight that the Balancing Responsibilities component exhibited insufficient internal consistency (α = 0.52). This low reliability may be attributed to the differential endorsement of the items. Specifically, “Balancing working at my job with my academics” was endorsed by only 32.5% of the sample (which was also the lowest endorsed item on the PSSI), while “Balancing my extracurriculars with academics” was endorsed by 77.7% of the sample. These endorsement rates suggest that these stressors were not experienced consistently across the sample, leading to variability in how participants responded to the items. Endorsement of items at significantly different rates can weaken the correlations between them, which may explain the low internal consistency observed in this study [34].

The findings of additional components suggest the presence of unique stressors among Ontario post-secondary students that may differ from those experienced by students in other urban, and potentially rural, regions. The present study was conducted at the University of Toronto, which is ranked as Canada’s top university and currently holds the 25th position globally, with a GPA of 3.3 + required for students seeking admission [35]. These rankings reflect the competitive, high-achieving nature of the university, which may expose its students to additional stressors, such as difficulties in managing responsibilities and concerns about future success, compared to students at other institutions [36]. Furthermore, given that this study took place in Toronto, a city known for its high cost of living, including housing, food, and transportation expenses [37,38], the emergence of the Future Worrying and Balancing Responsibilities components may reflect regional differences, as these components include items related to concerns about buying a house, having children, and balancing work commitments with academic demands. These findings underscore the importance of conducting a PCA for the PSSI with different student samples. Although the overall dimensionality may be largely consistent, small differences may exist that are essential for capturing the varied stressors and experiences unique to different student samples. Some of these stressors may have also been amplified or brought to the forefront during the COVID-19 pandemic, highlighting the ongoing need to assess stressors among post-secondary students in the post-pandemic context [39].

Although the PSSI is a well-validated and evidence-informed tool, its length (46 items) may present challenges for large-scale survey implementation across campuses due to participant burden [40]. To address this concern, the validity and reliability of a shorter 14-item version, the Brief-PSSI, were recently assessed [17]. Findings from an exploratory factor analysis demonstrated that the Brief-PSSI retained key stressors related to academics, the learning environment, campus culture, interpersonal relationships, and personal challenges from the original PSSI, while significantly reducing the number of items and alleviating participant burden. The Brief-PSSI assesses 14 stressors, including those related to exams, academic workload, grades, lack of clarity in course instruction, faculty interactions, post-secondary adjustment, pressure to succeed, discrimination, relationship management, social pressures, meeting performance expectations, maintaining a healthy lifestyle, financial concerns, and future uncertainties. A comparison of the stressor components in the present study with the items from the Brief-PSSI reveals substantial overlap in the identified stressor areas, further supporting the validity of the PCA results from this study and demonstrating its consistency and applicability across student samples.

By identifying components of the PSSI, this study provides a foundation for future research to examine how specific stressors impact student mental health, offering a more targeted approach to understanding the effects of stress on psychological outcomes. This is the first study to investigate the associations between PSSI components and perceived stress, depressive symptoms, and anxiety symptoms. The moderate to high significant positive correlations between the PSSI components and the PSS-10 suggest strong construct validity, as both assess stress-related experiences. Furthermore, significant positive correlations with depressive and anxiety symptoms provide evidence of the predictive validity of the PSSI components, demonstrating their practical utility as reliable predictors of mental health outcomes in post-secondary students [41]. By understanding the specific stressors that students face on campuses and the perceived severity of these challenges, institutions can develop targeted prevention and intervention strategies that address students’ unique experiences and needs.

### Limitations and future directions

The present study extends findings from Manitoba to a different province (i.e., Ontario); however, further research is needed to assess the generalizability of these results across Canada. Future studies should examine the dimensionality of the PSSI among students in other provinces and within multi-province Canadian samples to account for the diverse and region-specific stressors that students may experience. It may also be important to examine the PSSI across different types of institutions, as the post-secondary environment can vary significantly even within the same region. Differences in institutional size, research intensity, program specialization, available resources, and campus culture may all influence how students experience and report stressors. Although our sample included a more diverse ethnic composition than is typical and focused on first-year students, a group known to experience heightened stress during the transition to university [42], the sample was predominantly composed of students who were mostly women, White, East Asian, and South Asian. The Manitoba sample also had a similar gender composition. Prior studies have indicated that cisgender women and transgender women are more likely to report a greater number of stressors and to perceive these stressors as more severe, which could have contributed to some of the current findings [24,43]. As such, the generalizability of our findings to institutions with more diverse student samples, particularly those including men and non-binary individuals, may be limited. Further research is needed to assess the dimensionality of the PSSI across more diverse student samples, including men, non-binary students, international students, and graduate students, to determine whether the extracted components of the PSSI perform consistently across these groups.

The topic of the study, the use of convenience sampling, and the use of cash incentive may have introduced potential sources of bias that should be acknowledged. For example, self-selection bias may have occurred in the present study, as participants who were already experiencing stressors or had a particular interest in the study topic may have been more likely to volunteer. That said, the study was framed as a study on understanding student’s transition to university. In addition, the omission of items from the CESD-R and PSS-10 scales may have impacted the construct validity of these measures. Previous research emphasizes the importance of carefully considering the effects of item removal on both the validity and reliability of a construct [44]. However, shortened versions of these assessments have been used in prior work, and shown to have good construct validity [28,45]. Further, the internal consistency for both scales remained adequate in the present study. Nevertheless, future research utilizing the full versions of these measures is necessary to validate and corroborate the present findings.

The present findings also underscore the importance of conducting a PCA before applying previously established PSSI components from other studies to new student samples in different regions. Although there were notable similarities between the present study, the PCA conducted at the University of Manitoba [21], and the Brief-PSSI [17], slight differences emerged, such as the dropping of different items, variability in item loadings onto components, and the emergence of additional components. These differences suggest that the dimensionality of the PSSI may vary across student samples. It is also important to consider how the removal of items when creating components may impact the coverage and interpretability of the components themselves. For example, in the present study, four low-loading stressor items were excluded, which may have slightly reduced the breadth of stressors captured; however, with 42 out of 46 stressors retained, the resulting components still assess a wide range of stressors relevant to the present post-secondary student sample. Nevertheless, future research should conduct a PCA when applying the PSSI to new student samples to ensure the accuracy and validity of the extracted components and carefully consider the impact of any items excluded or retained within each unique context.

## Conclusion

In the present study, 12 principal components were identified, encompassing 42 of the 46 original PSSI items, with most components demonstrating moderate to high internal consistency. While notable similarities were observed between the dimensionality of the PSSI in the current study, the Manitoba PCA study, and the Brief-PSSI, some differences emerged, such as the identification of additional components, shifts in item loadings across components, and the exclusion of certain items. These variations highlight the importance of testing the measure’s dimensionality across diverse student samples. Additionally, by structuring the PSSI into distinct components, this study is the first to explore the associations between the identified stressor components and mental health indices. The results revealed that the PSSI components were generally moderately to strongly correlated with perceived stress, depressive symptoms, and anxiety symptoms, further validating their use as reliable predictors of mental health indices among post-secondary students. Future research should continue to validate the PSSI’s dimensionality across various university student samples to ensure its accuracy and broad applicability in campus-based mental health research.

## Supporting information

S1 Appendix Comparison of principal components and item loadings across Ontario and Manitoba samples. (DOCX)
