# Supplementary material for: A principal component analysis of the post-secondary student stressors index in a sample of Ontario students
Source: PLOS Ment Health. 2025 Sep 10;2(9):e0000431. doi: 10.1371/journal.pmen.0000431 (PMC12798515; doi:10.1371/journal.pmen.0000431)
Supplement: S1 Appendix — (DOCX) [file pmen.0000431.s001.docx]

**Appendix A.**

Comparison of principal components and item loadings across Ontario and Manitoba samples.

| **Ontario** | **Manitoba** |
| --- | --- |
| Academic Comparisons |  |
| 1. Comparing myself to others | 1. Comparing myself to others |
| 1. Feeling like my peers are smarter than I am | 1. Feeling like my peers are smarter than I am |
| 1. Feeling like I’m not working hard enough | 1. Feeling like I’m not working hard enough |
| 1. Comparing my life to others on social media | 1. Comparing my life to others’ on social media |
| 1. Meeting other peoples’ expectations of me | 1. Meeting people’s expectations of me |
| 1. Academic competition among my peers | 1. Academic competition among my peers |
| 1. Pressure to succeed | 1. Pressure to succeed |
| 1. Meeting my own expectations | 1. Meeting my own expectations |
|  | 1. Worrying about getting into a new program after graduating |
| Thesis Achievements |  |
| 1. Meeting my thesis/placement supervisor's expectations | 1. Meeting my thesis or placement supervisor’s expectations |
| 1. Lack of mentoring from my thesis/placement supervisor | 1. Lack of mentoring from my thesis or placement supervisor |
| 1. Working on my thesis | 1. Working on thesis |
| 1. Performing well at my professional placement (i.e., practicum, clerkship, etc.) | 1. Performing well at my professional placement (i.e., practicum, clerkship, etc.) |
| Interpersonal Relationships |  |
| 1. Making new friends | 1. Making new friends |
| 1. Maintaining friendships | 1. Maintaining friendships |
| 1. Networking with the “right” people | 1. Networking with the “right people” |
| 1. Feeling pressured to socialize | 1. Feeling pressure to socialize |
| 1. Balancing a social life with academics |  |
| Finances |  |
| 1. Worrying about paying off debt | 1. Working on paying off debt |
| 1. Having to take student loans | 1. Having to take student loans |
| Professor/Advisor Interactions |  |
| 1. Unclear expectations from professor | 1. Unclear expectations from professor |
| 1. Poor communication from professor | 1. Poor communication from professor |
| 1. Lack of guidance from professor | 1. Lack of guidance from professor |
| Exams |  |
| 1. Writing multiple exams around the same time | 1. Writing multiple exams on the same day |
| 1. Writing exams | 1. Writing exams |
| 1. Exams worth more than 50% of course grade | 1. Exams worth 50% or more of the final grade |
| 1. Preparing for exams | 1. Preparing for exams |
|  | 1. Receiving a bad grade |
| Healthy Lifestyle |  |
| 1. Making sure that I get enough exercise | 1. Making sure that I get enough exercise |
| 1. Making sure that I eat healthy | 1. Making sure that I eat healthy |
| 1. Making sure that I get enough sleep | 1. Making sure that I get enough sleep |
| 1. Having to prepare meals for myself | 1. Having to prepare meals for myself |
|  | 1. Balancing working at my job with my academics |
|  | 1. Balancing my extracurriculars with academics |
| Discrimination/Harassment |  |
| 1. Discrimination on campus | 1. Discrimination on campus |
| 1. Sexual harassment on campus | 1. Sexual harassment on campus |
| Post-Secondary Adjustment |  |
| 1. Adjusting to my program | 1. Adjusting to my program |
| 1. Adjusting to the post-secondary lifestyle | 1. Adjusting to the post-secondary lifestyle |
| Academic Workload |  |
| 1. Having multiple assignments due around the same time | 1. Having multiple assignments due around the same time |
| 1. Heavily weighted assignments | 1. Heavily weighted assignments |
| 1. Managing my academic workload | 1. Managing my academic workload |
| Balancing Responsibilities |  |
| 1. Balancing working at my job with my academics | Not Applicable |
| 1. Balancing my extracurriculars with academics |  |
| Future Worrying |  |
| 1. Worrying about getting into a new program after graduating | Not Applicable |
| 1. Worrying about reaching major “life events” (i.e., buying a house, marriage, children) |  |
| 1. Worrying about getting a job after graduating |  |

***Notes.*** Items that did not load onto components for the Ontario sample include: meeting with my professor, feeling guilty about taking time for my hobbies/interests, managing a high GPA, and receiving a bad grade. For the Manitoba sample, items that did not load onto components include: maintaining a high GPA, meeting with my professor, balancing a social life with academics, feeling guilty about taking time for my hobbies/interests, worrying about getting a job after graduating, and worrying about reaching major “life events” (i.e., buying a house, marriage, children). Items from both samples did not load above the 0.4 threshold.
